# Prevalence and Predictors of Overweight and Obesity Among Incarcerated Individuals in Saudi Arabia: A Cross-Sectional Study

**DOI:** 10.7759/cureus.89121

**Published:** 2025-07-31

**Authors:** Turki Bafaraj, Adnan Sardar, Aktham Alhawarat, Ramzi Shiku, Ahmed Jawi, Yousef Alzahrani, Ali Khaswi, Bander Agdi, Abdullah Alshareef, Shaimaa Malibari, Najim Z Alshahrani

**Affiliations:** 1 Directorate of Medical Services, Ministry of Interior, Jeddah, SAU; 2 General Directorate of Prisons Health, General Directorate of Medical Services, Ministry of Interior, Makkah, SAU; 3 Department of Family and Community Medicine, Faculty of Medicine, University of Jeddah, Jeddah, SAU

**Keywords:** correctional medicine, incarceration, nutritional behavior, obesity, physical activity, prison health, saudi arabia

## Abstract

Background

Weight gain is a common challenge in correctional settings, where individuals often face environmental and behavioral constraints that increase their vulnerability to obesity. Incarcerated populations are exposed to limited opportunities for physical activity, poor nutritional options, high levels of psychological stress, and the potential side effects of medications, all of which may contribute to weight gain. Despite these risks, obesity among prisoners remains an under-researched area in Saudi Arabia.

Methods

A cross-sectional analytical study was conducted among 388 incarcerated individuals in correctional facilities in Makkah City between February and April 2025. Participants were selected through systematic random sampling, including only those incarcerated for six months or longer. Data were collected using a structured questionnaire covering sociodemographic characteristics, dietary patterns, physical activity, stress levels, and behavioral factors. Body mass index (BMI) was calculated using standardized measurements of height and weight. Statistical analyses, including chi-square tests and multivariable logistic regression, were performed using R (version 4.3.0) within the RStudio environment (R Foundation for Statistical Computing, Vienna, Austria).

Results

Of the 388 participants, 270 (69.6%) were classified as overweight or obese. Weight gain was significantly associated with older age (median 40 vs. 36 years, p < 0.001), limited access to physical activity (24/270 (8.9%) vs. 66/118 (56.0%), p < 0.001), frequent high-calorie food intake (136/270 (50.4%) vs. 27/118 (22.9%), p < 0.001), and elevated stress levels (median score 7 vs. 5, p < 0.001). Emotional eating was more common among individuals who gained weight (75/270 (27.8%) vs. 20/118 (17.0%), p = 0.02). In contrast, current smoking was inversely associated with weight gain (197/270 (73.0%) vs. 100/118 (84.7%); adjusted odds ratio (AOR) = 0.40, 95% CI: 0.18-0.84). Accessibility to exercise facilities demonstrated the strongest protective effect (24/270 (8.9%) vs. 66/118 (56.0%); AOR = 0.065, p < 0.001).

Conclusion

Obesity is highly prevalent among incarcerated individuals in Saudi Arabia and is influenced by a combination of environmental, psychological, and behavioral factors. Targeted interventions addressing physical activity, stress management, and nutrition are urgently needed in correctional settings.

## Introduction

Weight gain refers to an increase in body mass, most commonly resulting from a sustained positive energy balance, where caloric intake exceeds energy expenditure [1]. This surplus energy is stored primarily as body fat, although in some cases, weight gain may also result from muscle development or fluid retention [2]. When excessive and persistent, weight gain can lead to overweight or obesity, both of which carry substantial health risks [3]. Overweight is generally defined as a body weight higher than what is considered optimal for a given height. It is commonly assessed using the Body Mass Index (BMI), where a BMI between 25.0 and 29.9 indicates overweight [4]. Obesity, by contrast, is characterized by an abnormal or excessive accumulation of body fat, typically reflected by a BMI of 30.0 or higher [4]. While these classifications are widely used, they may not fully capture the complexities of body composition or health outcomes, especially in institutionalized settings.

The global burden of obesity has intensified significantly over recent decades. According to the World Health Organization (WHO), the prevalence of obesity has reached alarming levels in recent decades. In 2022, approximately one in eight individuals worldwide were living with obesity, and more than 2.5 billion adults were classified as overweight, including 890 million who were obese [5]. Since 1990, adult obesity has more than doubled, and adolescent obesity has increased fourfold, indicating a worsening trend that spans across age groups and regions [5]. The health implications of obesity are extensive and well-documented. It is strongly associated with a range of chronic illnesses such as type 2 diabetes, hypertension, coronary artery disease, stroke, and osteoarthritis [6]. These conditions significantly increase the risk of premature death and are estimated to reduce life expectancy by up to seven years [7]. Furthermore, obesity disproportionately affects individuals from disadvantaged socioeconomic backgrounds, exacerbating existing health inequities [8].

Saudi Arabia has not been immune to these global trends. Recent national statistics have shown alarming rates of overweight and obesity across different age groups. A 2024 report from the General Authority for Statistics (GASTAT) indicated that 23.1% of the population aged 15 and older were obese, while an additional 45.1% were overweight [9]. Among children aged 2 to 14, 14.6% were classified as obese and 33.3% as overweight. These figures reflect a significant and growing public health issue, with considerable implications for the nation's healthcare system, economy, and overall well-being.

Although much of the research and public health attention has focused on the general population, there is growing recognition that specific subgroups face distinct risk factors and health challenges. Among these, incarcerated individuals constitute a particularly vulnerable group. Before imprisonment, many inmates experience socioeconomic deprivation, unemployment, limited education, and unstable housing, all of which contribute to a higher burden of disease [10,11]. Once incarcerated, individuals face additional health challenges linked to the prison environment, including restricted autonomy, institutional diets, limited access to physical activity, and heightened psychological stress [12].

One of the most concerning health trends in correctional facilities is the rise in overweight and obesity among inmates [10]. Diet and physical activity, the two primary modifiable risk factors for weight gain, are largely regulated within prison settings [10]. While meals are typically provided by the institution, the nutritional quality can vary significantly, and in some contexts, inmates may supplement their diet with food purchased from prison canteens, which is often high in calories and low in nutritional value [10]. Physical activity levels are similarly constrained by the prison regime, which dictates both the availability and duration of exercise opportunities. Security protocols, staffing, and facility infrastructure often limit consistent access to physical activity [13].

Two key theories explain how incarceration influences prisoner health behaviors: deprivation and importation [10]. The deprivation theory suggests that the prison environment strips individuals of autonomy, possessions, and social connections, leading them to adapt to a distinct prison culture. This includes adopting a “tough” persona and striving for physical dominance, often by increasing body mass [14]. The importation theory, on the other hand, argues that inmates bring pre-existing behaviors and attitudes from their communities, so those with poor health habits continue them in prison [15]. Gender also shapes these patterns. Female prisoners show higher rates of disordered eating, while male prisoners may focus on weight gain for status [16]. A systematic review found that imprisoned women were more likely to be obese than women in the general population, whereas male inmates were generally less likely to be obese, except in the United States [17]. These differences suggest that imprisonment affects health behaviors in gender-specific ways.

Despite the global recognition of obesity as a public health priority, there remains a lack of data addressing weight-related health issues within correctional settings in the Middle East, particularly in Saudi Arabia. Most available research focuses on the general population, overlooking the unique environmental, psychological, and behavioral factors that influence weight gain during incarceration.

This study seeks to fill that gap by examining the prevalence and predictors of weight gain among incarcerated individuals in correctional facilities in Makkah City. We aimed to explore not only the extent of the problem but also the underlying lifestyle, environmental, and psychosocial contributors to weight gain within prison walls.

## Materials and methods

Study design and setting

A cross-sectional analytical study was conducted among incarcerated individuals in correctional facilities within Makkah City, Saudi Arabia, between February and April 2025. The study population comprised adult male and female prisoners who had been incarcerated for a minimum period of six months. This duration threshold was established to ensure adequate exposure to the prison environment and its potential influences on weight status. Individuals with severe medical conditions that could independently affect body weight, including diabetes mellitus, active malignancy, or advanced organ failure, were excluded from the study to minimize confounding variables.

Sample size calculation

The required sample size was estimated using OpenEpi (Version 3.01), based on an expected prevalence of overweight and obesity of 73.3% among incarcerated populations, as reported in a recent systematic review and meta-analysis [10]. Assuming a 95% confidence level, 5% absolute precision, and a design effect of 1.0, the minimum calculated sample size was 301 participants. To account for potential non-response and incomplete data, the target sample was increased by approximately 29%, resulting in a final sample size of 388 participants.

Sampling method

Participants were recruited through systematic random sampling from comprehensive prison rosters to minimize selection bias and ensure representative coverage across different demographic strata. This probabilistic sampling approach facilitated the generalizability of findings to the broader incarcerated population within similar correctional settings.

Data collection

A comprehensive, structured questionnaire was administered to collect detailed information across multiple domains. Data were collected through interviewer-administered, face-to-face structured interviews conducted by trained healthcare staff. Sociodemographic characteristics included age, gender, educational attainment, marital status, and duration of incarceration. Lifestyle factors encompassed smoking status, dietary patterns, including meal frequency and consumption of high-calorie foods and sugary beverages, and perceived quality of provided meals. Environmental and behavioral variables captured aspects of prison conditions such as facility overcrowding, availability of physical activity spaces, exercise accessibility and frequency, and stress levels during incarceration, which were assessed using the 10-item Perceived Stress Scale (PSS-10), a validated and widely used tool for measuring psychological stress. Coping mechanisms employed by participants were also recorded. The questionnaire was adapted from previously published studies in correctional health and underwent pilot testing for contextual relevance and clarity.

Anthropometric measurements (height and weight) were taken by trained nursing staff using standardized procedures and calibrated equipment. Body mass index (BMI) calculations were performed using these measurements. Weight status classifications followed World Health Organization guidelines, with participants categorized as underweight (BMI < 18.5 kg/m²), normal weight (BMI 18.5-24.9 kg/m²), overweight (BMI 25.0-29.9 kg/m²), or obese (BMI ≥ 30.0 kg/m²) [2]. For analytical purposes, participants were subsequently dichotomized into two groups: those with normal weight and those classified as overweight or obese, representing weight gain within the correctional environment.

Statistical analysis

All statistical analyses were performed using RStudio (version 4.3.0; R Foundation for Statistical Computing, Vienna, Austria). Descriptive statistics summarized demographic, lifestyle, and environmental characteristics. Categorical variables were reported as frequencies and percentages, while continuous variables were presented as medians and interquartile ranges due to non-normal distributions. Group comparisons were conducted using Pearson’s chi-squared tests for categorical variables and Wilcoxon rank-sum tests for continuous variables. Fisher’s exact test was applied when expected cell counts were below threshold. A multivariable logistic regression model identified independent predictors of weight gain, including variables significant in bivariate analysis. Adjusted odds ratios (ORs) with 95% confidence intervals (CIs) quantified associations. R packages, including tidyverse, dplyr, ggplot2, gtsummary, broom, and waffle, supported data handling and visualization. Graphical outputs included a waffle plot (barriers to exercise), a Sankey diagram (stress, coping, and BMI pathways), and a scatter plot (BMI vs. stress). A p-value <0.05 was considered statistically significant for all analyses.

Ethical considerations

The study was approved by the Institutional Review Board of Security Forces Hospital Medical City (IRB protocol: 0789-180125; registration: HAP-02-K-052) and was exempted from full review. Written informed consent was obtained from all participants after explaining the study’s purpose, procedures, risks, and benefits. Confidentiality was ensured through data anonymization, and participants could withdraw at any point without affecting their legal or service status. Measurement tools were regularly calibrated, and data entry underwent double verification to ensure accuracy and integrity.

## Results

Sociodemographic and lifestyle characteristics

Among the 388 incarcerated individuals included in the study, 69.6% (n = 270) were classified as overweight or obese, while 30.4% (n = 118) had normal weight based on BMI criteria (Table 1). Participants with weight gain were significantly older (median age: 40 vs. 36 years, p < 0.001) and less likely to be current smokers (73% vs. 85%, p = 0.01). No significant differences were observed in gender distribution (p = 0.38), educational attainment (p = 0.06), or marital status (p = 0.81). Those with weight gain were more likely to report eating more than three meals per day (14% vs. 5.1%, p = 0.05) and consuming high-calorie foods frequently (50% vs. 23%, p < 0.001). Sugary b

c5everage consumption was also significantly higher in this group (58% vs. 38%, p = 0.003).

**Table 1:** Sociodemographic and lifestyle characteristics of prisoners stratified by weight gain ^1^ n (%); Median (Q1, Q3), ^2^ Pearson’s Chi-squared test; Wilcoxon rank sum test; Fisher’s exact test.

Characteristics	Weight Gain		P-value^2^
	No N = 1181	Yes N = 2701	
Age	36 (29, 43)	40 (32, 46)	<0.001
Gender			0.38
Female	9 (7.6%)	31 (11%)	
Male	109 (92%)	239 (89%)	
Educational level			0.06
Higher Education	1 (0.8%)	1 (0.4%)	
No formal education	3 (2.5%)	23 (8.5%)	
Primary Education	96 (81%)	216 (80%)	
University	18 (15%)	30 (11%)	
Marital states			0.81
Divorced	19 (16%)	48 (18%)	
Married	29 (25%)	75 (28%)	
Single	68 (58%)	140 (52%)	
Widowed	2 (1.7%)	7 (2.6%)	
Incarceration duration			0.6
Less than 1 year	16 (14%)	44 (16%)	
1-5 years	45 (38%)	112 (41%)	
5-10 years	36 (31%)	68 (25%)	
More than 10 years	21 (18%)	46 (17%)	
Current Smoker	100 (85%)	197 (73%)	0.01
Meal frequency			0.05
One	3 (2.5%)	12 (4.4%)	
Two	38 (32%)	74 (27%)	
Three	71 (60%)	147 (54%)	
More than 3	6 (5.1%)	37 (14%)	
Quality of food provided			0.7
Excellent	5 (4.2%)	13 (4.8%)	
Fair	26 (22%)	51 (19%)	
Good	38 (32%)	79 (29%)	
Poor	49 (42%)	127 (47%)	
High-calorie food consumption			<0.001
Never	11 (9.3%)	14 (5.2%)	
Often	27 (23%)	136 (50%)	
Rarely	25 (21%)	35 (13%)	
Sometimes	55 (47%)	85 (31%)	
Snacks between meals			0.7
1-2 Per week	58 (49%)	120 (44%)	
3-5 Per week	39 (33%)	102 (38%)	
Daily	20 (17%)	45 (17%)	
Never	1 (0.8%)	3 (1.1%)	
Sugary beverages consumption			0.003
Never	16 (14%)	21 (7.8%)	
Often	45 (38%)	157 (58%)	
Rarely	21 (18%)	37 (14%)	
Sometimes	36 (31%)	55 (20%)	

Prison environment, physical activity, and stress

Limited access to physical activity spaces (18% vs. 30%, p = 0.009) and lower exercise accessibility (8.9% vs. 56%, p < 0.001) were more prevalent among participants with weight gain (Table 2). Stress levels were higher in this group (median score: 7 vs. 5, p < 0.001), and emotional eating was more frequent (28% vs. 17%, p = 0.02). Exercise frequency differed significantly; none of the weight-gain group exercised daily, compared to 7.6% in the normal-weight group (p < 0.001).

**Table 2:** Association between prison conditions, inmate behaviors, and weight gain ^1 ^n (%); Median (Q1, Q3), ^2^ Pearson’s Chi-squared test; Wilcoxon rank sum test; Fisher’s exact test.

Characteristics	Weight Gain		P-value^2^
	No N = 118^1^	Yes N = 270^1^	
Space for Physical Activity	35 (30%)	48 (18%)	0.009
Overcrowding facility			0.6
Moderately crowded	25 (21%)	54 (20%)	
Not crowded	20 (17%)	46 (17%)	
Slightly crowded	27 (23%)	49 (18%)	
Very crowded	46 (39%)	121 (45%)	
Eating more when stressed	20 (17%)	75 (28%)	0.02
Cope with stress			0.2
Eating	10 (8.5%)	42 (16%)	
Exercising	6 (5.1%)	15 (5.6%)	
Other	29 (25%)	63 (23%)	
Sleeping	73 (62%)	150 (56%)	
Stress levels during incarceration	5 (3-6)	7 (6-9)	<0.001
Exercise accessibility	66 (56%)	24 (8.9%)	<0.001
Activity type			0.2
Other	28 (24%)	40 (15%)	
Team sports	13 (11%)	29 (11%)	
walking	70 (61%)	180 (69%)	
Walking	4 (3.5%)	10 (3.9%)	
Weight lifting	28 (24%)	40 (15%)	
Unknown	11	4	
Exercise Frequency			<0.001
1-2 Per week	68 (58%)	190 (70%)	
3-5 Per week	38 (32%)	62 (23%)	
Daily	9 (7.6%)	0 (0%)	
Never	3 (2.5%)	18 (6.7%)	

Multivariate analysis of predictors of weight gain

Logistic regression analysis (Table 3) identified several independent predictors. Smoking was associated with lower odds of weight gain (AOR = 0.40, 95% CI: 0.18-0.84, p = 0.020). Frequent consumption of high-calorie foods nearly tripled the odds (AOR = 3.06, 95% CI: 0.96-9.69, p = 0.056), although this narrowly missed statistical significance. Each unit increase in stress score was associated with a 28% increase in the odds of weight gain (AOR = 1.28, 95% CI: 1.14-1.44, p < 0.001). Exercise accessibility had the strongest protective effect (AOR = 0.065, 95% CI: 0.030-0.133, p < 0.001).

**Table 3:** Multivariate logistic regression analysis for weight gain risk factors.

Factors	Adjusted OR	95% CI Low	95% CI High	P-value
(Intercept)	1.07	0.147	8.27	0.951
Smoking: Yes	0.402	0.182	0.844	0.0196
High-calorie food: Often	3.06	0.959	9.69	0.0562
Space for Physical Activity: Yes	2.00	0.933	4.55	0.0847
Stress levels during incarceration	1.28	1.14	1.44	<0.0001
Exercise accessibility: Yes	0.065	0.0297	0.133	<0.0001

Barriers to physical activity

**Figure 1:**
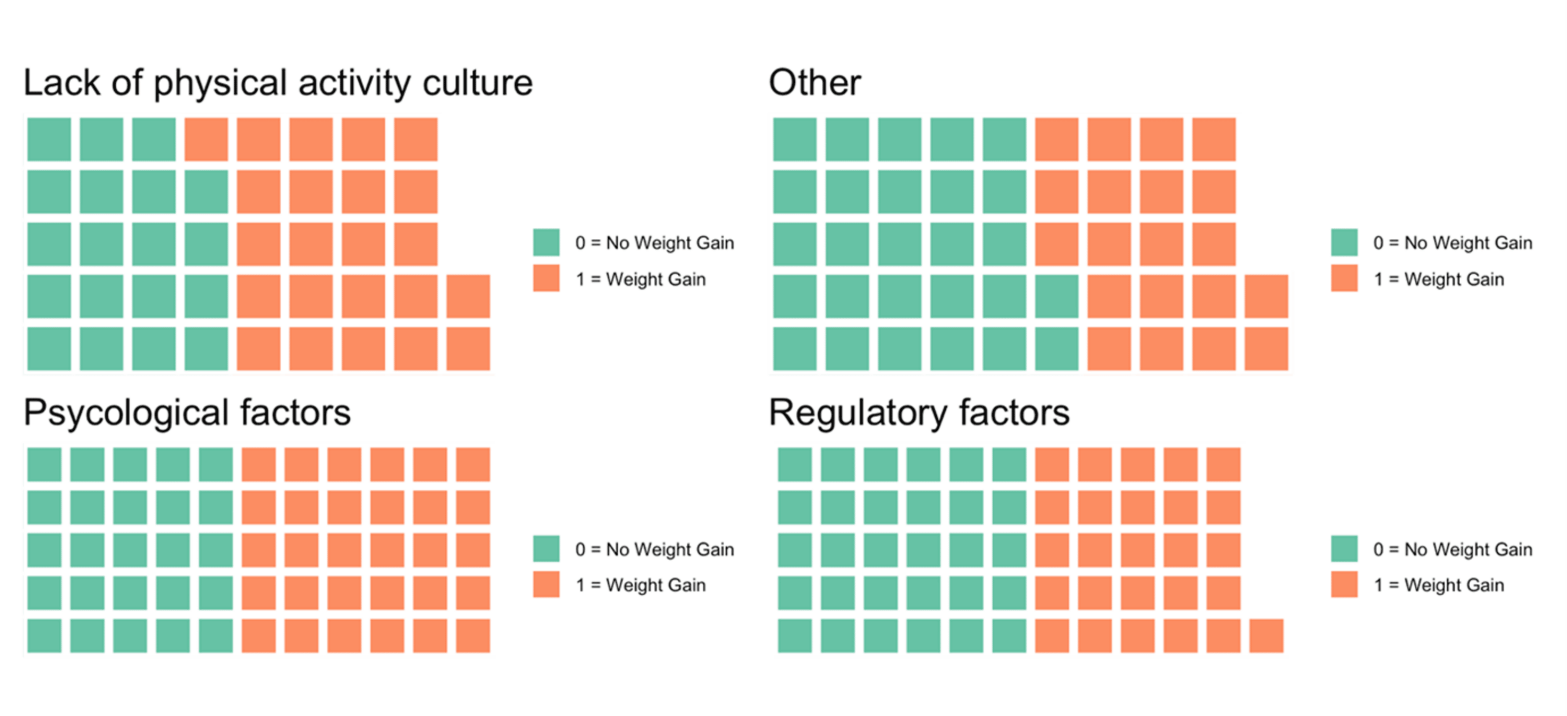
Waffle Plot visualization of barriers to exercise among individuals with and without weight gain The ‘Other’ category included barriers such as fear of injury, overcrowded conditions, and limited access to designated exercise areas.

As illustrated in the waffle plot (Figure 1), psychological barriers were the most frequently reported obstacle to exercise among those with weight gain (30.4%), followed by regulatory restrictions (26.3%), lack of physical activity culture (23%), and other barriers (20.4%). The ‘Other’ category included barriers such as fear of injury, overcrowded conditions, and limited access to designated exercise areas. In contrast, individuals without weight gain cited regulatory barriers most often (29.7%), with fewer reporting psychological (24.6%) and cultural (18.6%) obstacles.

Stress, coping strategies, and BMI outcomes

**Figure 2:**
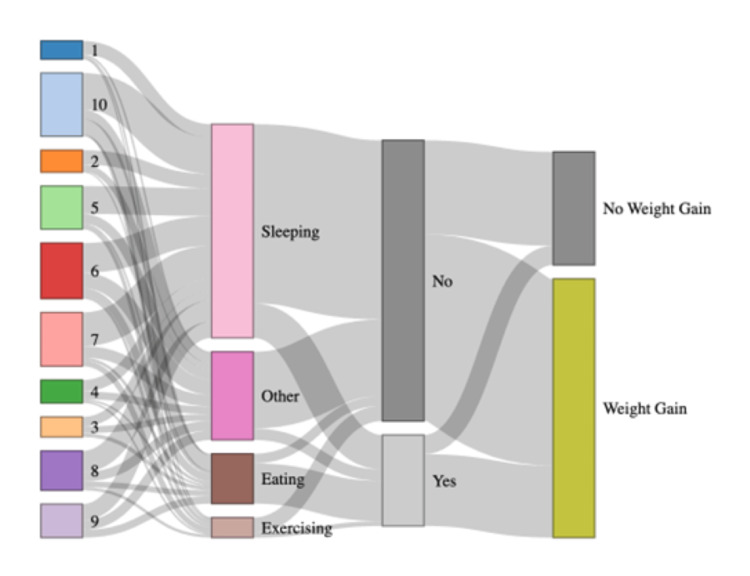
Sankey diagram depicting the behavioral pathway from stress level during incarceration to coping strategy, emotional eating, and BMI classification Items 1 to 10 represent contributors to weight gain during incarceration: (1) stress, (2) emotional eating, (3) poor diet, (4) low physical activity, (5) limited healthcare access, (6) poor sleep, (7) mental health conditions, (8) social isolation, (9) medication side effects, and (10) boredom. 'Other' comprises peer influence, cultural norms, and limited access to healthier food alternatives. 'Eating' represents emotional eating.

The Sankey diagram (Figure 2) demonstrates the pathway from stress levels to coping behaviors, emotional eating, and BMI status. Emotional eating was more common among those who used food to cope with stress, contributing to higher weight gain (n = 75). However, even among those who denied emotional eating, weight gain remained prevalent (n = 195), indicating additional contributing factors. Sleeping was the dominant coping strategy across groups and was associated with lower emotional eating, but did not appear to prevent weight gain.

**Figure 3:**
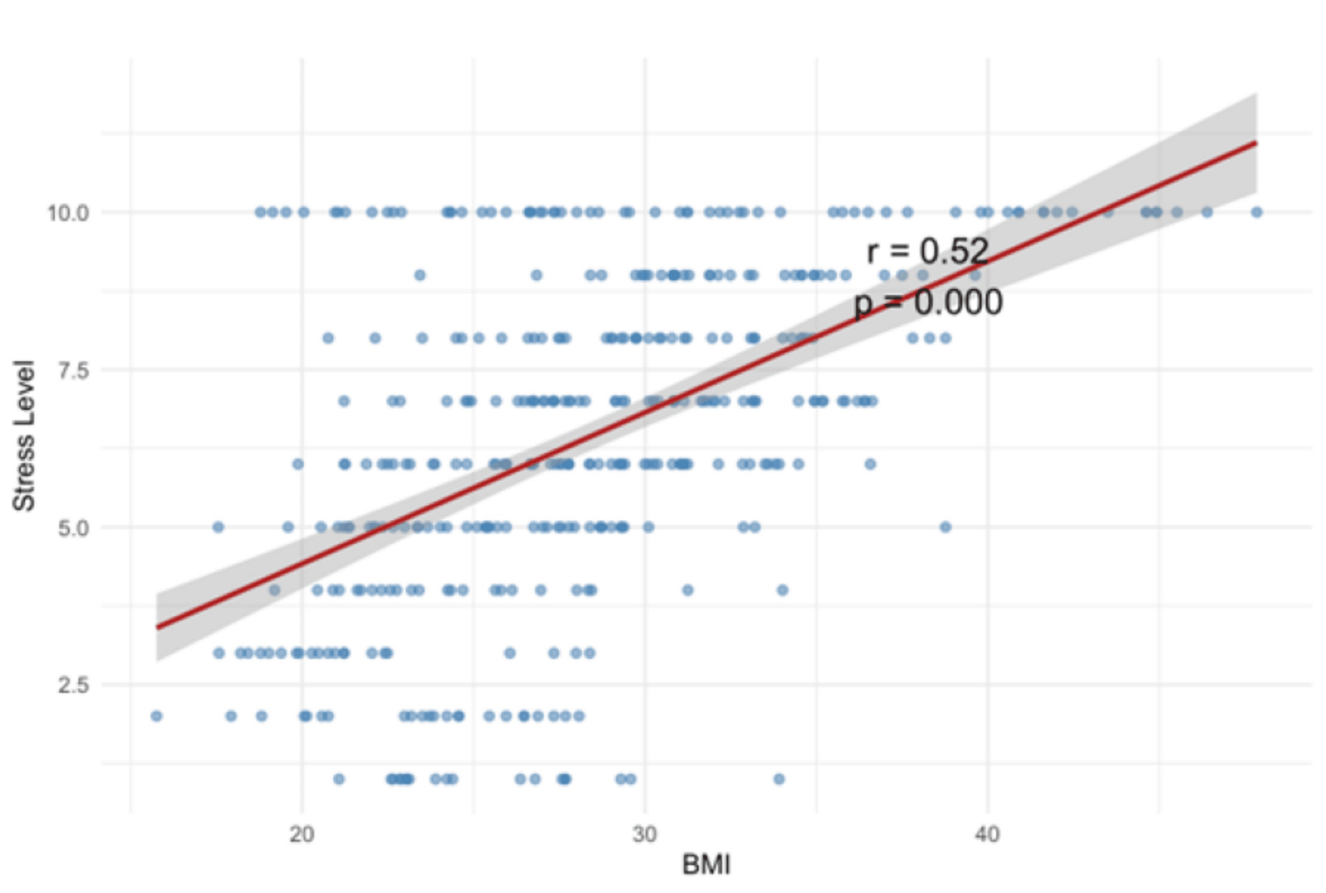
Scatter Plot of Body Mass Index (BMI) and stress levels experienced during incarceration among the incarcerated cohort

The scatter plot (Figure 3) illustrates the positive association between stress levels and BMI, highlighting a trend of increasing body mass with higher perceived stress during incarceration (r=0.52, P-value < 0.001).

## Discussion

This study aimed to examine the prevalence of weight gain among incarcerated individuals in Makkah and to identify associated behavioral, psychological, and environmental risk factors. To our knowledge, this is the first study in Saudi Arabia to systematically investigate weight gain among prisoners using a structured analytical approach. We observed a notably high prevalence of weight gain (69.6%) compared to normal weight (30.4%). This aligns with findings from a systematic review and meta-analysis reporting weight gain as a common outcome during incarceration [10]. Similarly, a U.S.-based study reported comparable prevalence rates of obesity (38%) and overweight (40%) among prisoners [18]. The slightly higher rates observed in our population may be attributed to distinct dietary habits and limited health-promoting infrastructure within correctional facilities in the region, as noted in a study examining barriers to healthy eating in Arab societies [19].

In contrast, general population data from Saudi Arabia reported lower prevalence rates of overweight (32.8%) and obesity (23%) [20], highlighting the prison environment as a distinct context with heightened vulnerability to weight gain. Age emerged as a significant factor, with individuals who gained weight being older on average than those who maintained a normal weight (median age 40 vs. 36 years). This finding suggests that older incarcerated individuals may be more susceptible to weight gain, possibly due to age-related metabolic changes, reduced physical activity, or cumulative stress exposure [21,22]. Further research is needed to explore the mechanisms behind this association within correctional settings.

Interestingly, individuals with weight gain were less likely to be current smokers, a finding consistent with U.S. data indicating a potential protective relationship between smoking and body weight [23]. However, this relationship is complex and should not be interpreted as a justification for smoking to manage weight. While nicotine can suppress appetite and temporarily increase metabolic rate, leading to a lower body mass index (BMI), the health consequences of smoking are severe [24]. These include increased risks of cardiovascular disease, respiratory illness, and various cancers [25]. Although smoking cessation is often associated with post-cessation weight gain, it remains a critical public health priority because of its significant long-term health benefits. These findings underscore the importance of developing comprehensive interventions that support both smoking cessation and healthy weight management within correctional environments.

Contrary to some international findings that reported higher weight gain prevalence among incarcerated females [26], our results showed no significant gender differences, which is supported by studies focusing solely on female prison populations [27]. Access to physical activity was notably reduced among individuals with weight gain, consistent with prior research from Saudi Arabia linking a lack of exercise opportunities to increased obesity risk in confined settings [28].

Emotional eating was more common among those with weight gain (28% vs. 17%), echoing a UK-based study that described disordered eating behaviors among female inmates [29]. Stress emerged as a key factor, with each unit increase in reported stress levels significantly associated with higher odds of weight gain. This finding aligns with physiological evidence suggesting elevated cortisol levels under chronic stress may contribute to abdominal fat accumulation [30].

Barriers to exercise were diverse and often overlapping. Among individuals who gained weight, psychological factors were the most commonly cited barrier (30.4%), followed by regulatory constraints (26.3%) and lack of a supportive physical activity culture (23%). This pattern highlights the central role of psychological distress in limiting engagement with exercise, consistent with findings from a Turkish study that reported a stress prevalence of 52.4% among incarcerated individuals [31]. Structural limitations were also significant, with regulatory barriers pointing to deficiencies in prison policies, restricted access to facilities, and a lack of programmatic support, as documented in prior research on prison health systems [32].

Notably, among those without weight gain, regulatory issues were more frequently reported (29.7%), while psychological factors were less dominant. This contrast suggests that psychological stress may exert a stronger influence on behavioral pathways contributing to weight gain [33]. The relatively low recognition of physical activity as a health-promoting behavior in both groups, particularly among those who gained weight, underscores a broader problem of limited health literacy and the absence of an institutional culture that encourages physical engagement. These findings call for more than infrastructure improvements; they require integrated interventions that address mental health, remove policy-related obstacles, and promote physical activity as a fundamental component of prisoner well-being.

To manage psychological distress, prisoners adopt various coping mechanisms. Emotional eating was a prominent strategy linked to weight gain, as reported in earlier studies [29]. However, a substantial proportion of those who did not engage in emotional eating still experienced weight gain, indicating that weight gain in prison settings is multifactorial. Environmental, behavioral, and physiological contributors must also be considered [27,34].

Given the well-documented health risks associated with excess weight, including metabolic disorders and increased morbidity, the implementation of structured obesity interventions in correctional facilities is both necessary and actionable. Evidence from prior research demonstrates that targeted interventions addressing diet, physical activity, and psychological well-being can significantly improve health outcomes in incarcerated populations [35]. These efforts should move beyond awareness campaigns and include systemic changes such as employing registered dietitians to tailor meal plans to individual nutritional needs and assigning qualified fitness instructors to oversee structured physical activity programs. Equally important is the integration of mental health professionals within prison health services. Given the high prevalence of psychological distress among prisoners [31,36,37], addressing mental health is not optional but fundamental. Incorporating mental health care can reduce maladaptive coping mechanisms such as emotional eating and contribute to sustainable weight management and improved overall health in correctional environments.

Limitations

This study has several limitations that should be considered when interpreting the findings. The cross-sectional design restricts the ability to establish causal relationships between identified risk factors and weight gain. Data on dietary habits, stress levels, and physical activity were self-reported, which may introduce recall or social desirability bias. Stress-related and psychological data were based on self-rating, not validated scales. While efforts were made to recruit a representative sample through systematic random sampling, the study was conducted in correctional facilities within a single city, which may limit generalizability to other regions or prison systems. Additionally, unmeasured confounders such as pre-incarceration weight history or specific medication use may have influenced weight outcomes but were not accounted for in the analysis.

## Conclusions

This study highlights a high prevalence of overweight and obesity among incarcerated individuals in Makkah, with nearly seven in ten participants affected. Weight gain was significantly associated with modifiable factors, including limited access to exercise, frequent consumption of high-calorie foods, and elevated stress levels. While smoking showed a protective association, the underlying mechanisms warrant further investigation. The findings emphasize the importance of addressing structural and behavioral contributors to weight gain within correctional settings. Interventions that promote physical activity, improve dietary quality, and offer psychological support may help mitigate obesity risk and improve health outcomes for incarcerated populations. These results provide a foundation for future longitudinal research and targeted policy development in prison health systems.
